# Association of high waist-to-height ratio with functional outcomes in patients with acute ischemic stroke: A report from the ACROSS-China study

**DOI:** 10.1097/MD.0000000000006520

**Published:** 2017-03-31

**Authors:** Ping Yu, Yuesong Pan, Huaguang Zheng, Xianwei Wang, Hongyi Yan, Xu Tong, Jing Jing, Xiao Zhang, Li Guo, Yilong Wang

**Affiliations:** a Department of Neurology, Second Hospital, Hebei Medical University, Shijiazhuang, Hebei Province; b Department of Neurology, Tiantan Clinical Trial and Research Center for Stroke, Beijing Tiantan Hospital, Capital Medical University; c Beijing Key Laboratory of Translational Medicine for Cerebrovascular Disease; d China National Clinical Research Center for Neurological Diseases; e Vascular Neurology, Department of Neurology, Beijing Tiantan Hospital, Capital Medical University, Beijing; f Department of Neurology, Tangshan Gongren Hospital, Hebei Medical University, Tangshan; g Hand Surgery Department, Third Hospital, Hebei Medical University, Shijiazhuang, Hebei Province, China.

**Keywords:** abdominal adiposity, acute ischemic stroke, functional recovery, mortality, waist-to-height ratio

## Abstract

The aim of our study was to investigate the relationship between the waist-to-height ratio (WHR) and all-cause mortality and functional outcomes after acute ischemic stroke in a prospective cohort study.

A total of 2076 patients (36.66% females) with ischemic stroke were analyzed from ACROSS-China, which is a nationwide, prospective, hospital-based stroke registry aimed to detect the glucose abnormality in China. One-year follow-up evaluation was done by telephone interview. Outcome measures were all-cause mortality and functional outcome defined as modified Rankin score being 6 and from 0 to 6, respectively. We identified predictors for functional outcomes using logistic regression analysis, and mortality outcome using Cox proportional hazards model which incorporated covariates with *P* value of < 0.2 in the univariate analysis and those of clinical importance.

The higher WHR was associated with worse functional outcome, but not predictive of the patients’ mortality outcomes. Compared with the first quartile (≤0.48), the fourth quartile of the WHR was more likely to be associated with poor functional recovery (fourth quartile (≥0.56), OR = 1.38, CI: 1.08–1.77, *P* = 0.01; third quartile OR = 1.10, CI: 0.86–1.40, *P* = 0.45; second quartile OR = 1.05, CI: 0.83–1.33, *P* = 0.71).

Our findings suggest that abdominal fat accumulation may be associated with functional recovery after stroke, and is not associated with mortality after stroke. Compared with the lowest quartile, the highest quartile of WHR at admission was possibly associated with worse postacute ischemic stroke functional recovery.

## 1 Introduction

Obesity is a major public health issue and is a modifiable risk factor for stroke.^[1]^ But numerous studies have shown an inverse relationship between obesity (or being overweight) and poststroke mortality. This phenomenon was called the “stroke obesity paradox”, the reason of which is still controversial.^[2–5]^

The proxy of most of the studies previously conducted was body mass index (BMI). It is an index of general obesity, not taking into account the distribution of body fat. However, people with regional accumulation of fat in abdominal area were more likely to have a worse metabolic profile than the general obese people.^[6]^ And studies showed abdominal obesity is correlated with the incidence of stroke, especially ischemic stroke.^[7–9]^ In the Kailuan study, which was a prospective population-based cohort study in the Kailuan community in China, we observed that every measurement of adiposity was only associated with the risk for total stroke and ischemic stroke, but not for hemorrhagic stroke. This is possibly explained by associations between obesity measures and ischemic stroke incidence was largely explained by mediators related to obesity, for example, diabetes mellitus and dyslipidemia. Therefore, it is necessary for us to further explore the relationship of the extent of abdominal fat accumulation and the mortality after ischemic stroke to see if the paradox still existed in this circumstance.

Recent studies have found that the waist-to-height ratio (WHR) is a simple and applicable index of abdominal obesity.^[10,11]^ And it displayed some advantages over other indexes such as waist circumference and the waist-to-hip ratio (WHpR).^[9,12,13]^ The acute stroke across China trial (ACROSS-China) was a nationwide multicenter prospective stroke registry conducted between 2007 and 2008. Our study aimed to investigate the prognostic value of the WHR on 1-year mortality and functional outcomes after ischemic stroke using the patients in this registry.

## 2 Methods

### 2.1 Study population

The study patients in the study were from the ACROSS-China trial. The ACROSS-China was a nationwide, prospective, multicenter cohort study to determine the prevalence of glucose metabolism abnormality in patients with first ever stroke. The inclusion and exclusion criteria for ACROSS-China have been published elsewhere.^[14]^

Patients were excluded if they had the following conditions: intracerebral or subarachnoid hemorrhage; lack of baseline waist circumference or body height information; lack of 1-year follow-up modified Rankin scale (mRS) score^[15,16]^ information.

Approval was obtained from the ethics committee of all participating centers, and all patients or their designated family members gave written informed consent.

### 2.2 Data collection

All of the baseline data were obtained within 24 hours after admission. Demographics such as age, gender, and traditional vascular risk factors, including history of atrial fibrillation, coronary heart disease, heart failure, hypertension, hyperlipidemia, and diabetes mellitus as well as current smoking and moderate and heavy drinking, were recorded. “Current smoking” was defined as an individual who smoked at the time of stroke. “Moderate and heavy drinking” means ≥2 standard alcoholic beverages consumed per day. Other variables included in our study were as follows: systolic blood pressure, diastolic blood pressure, fasting blood glucose and HbA1C level, serum triglycerides, total cholesterol, low-density lipoprotein cholesterol, high-density lipoprotein cholesterol, and creatinine. The severity of neurological impairment was evaluated at admission using the National Institutes of Health Stroke Scale (NIHSS).^[17]^ After the index event, secondary prevention treatments were administered, including antiplatelet, anticoagulant, antihypertensive, and lipid-lowering therapies. So in-hospital oral hypoglycemic and insulin drugs, antithrombotic drugs, antihypertension drugs, and lipid-lowering drugs were recorded.

Twelve months after admission, follow-up interviews were conducted via telephone by trained research personnel at Beijing Tiantan Hospital using a standardized script. The caregiver was contacted and interviewed when the information provided by the patient was insufficient.

All participants were shoeless when their body heights were measured, and waist circumference was measured at the level of the umbilicus. Both were measured to the nearest 0.1 cm and were assessed by trained medical staff. The WHR was calculated as waist circumference divided by body height.

### 2.3 Outcome measures

The acute ischemic stroke patients were classified according to the Trial of Org 10,172 Acute Stroke Treatment system^[18]^ and were assessed 365 ± 7 days after stroke onset to obtain the functional outcomes and mortality outcomes. mRS scores obtained via telephone were used to determine the functional outcomes of the patients, and these scores were graded from 0 to 6. Patients who died were given a mRS score of 6, while the mRS scores of surviving patients ranged from 0 to 5.

### 2.4 Statistical methods

We used SAS software, version 9.4 (SAS Institute Inc, Cary, NC) for analysis. *χ*^2^, *t* tests or rank sum tests were used to determine differences in clinical characteristics among patients with different grades of functional outcomes, or with survival outcomes and death outcomes. We identified predictors for functional outcomes using logistic regression analysis, and mortality outcome using Cox proportional hazards model which incorporated covariates with *P* value of <0.2 in the univariate analysis and those of clinical importance. The WHR was classified into quartiles and included in the logistic regression model and Cox proportional hazards model for further investigation. In addition, sex- and age-based subgroup studies were conducted. Two-sided *P* values were reported for all analyses. Values of *P* < 0.05 were considered statistically significant.

## 3 Result

**Figure 1:**
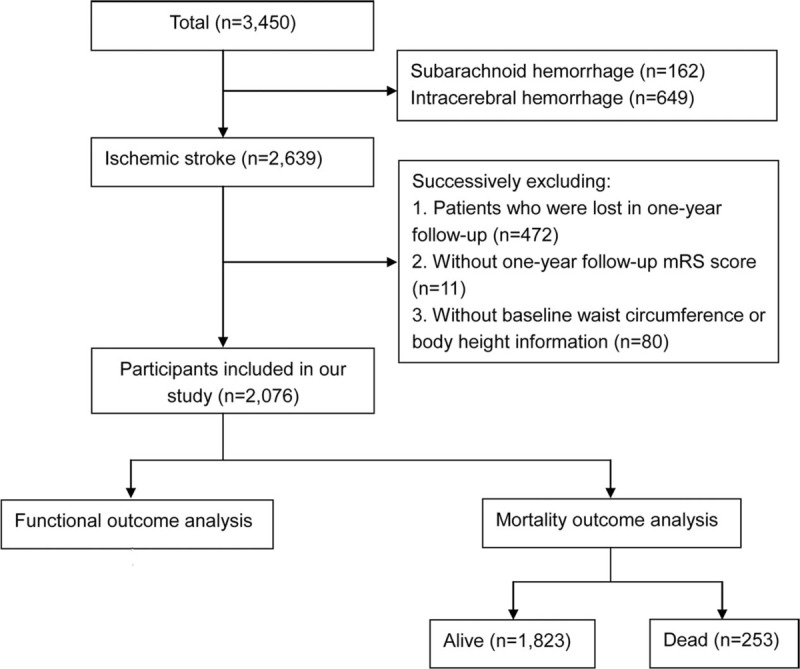
Flowchart of patient selection.

The ACROSS-China study included 3450 Chinese patients between 2008 and 2009. According to our inclusion and exclusion criteria, we excluded 811 participants who had hemorrhagic strokes, 472 participants who were lost at the 1-year follow-up, 11 participants who lacked a 1-year follow-up mRS score, and 80 participants without baseline waist circumference or body height information. A total of 2076 patients were included in the study (Table 1) (Fig. 1). The baseline characteristics of the participants without 1-year follow-up and those included in our study were similar, except a higher proportion of diabetes mellitus in the past history among the participants included in our study (appendix 1).

**Table 1:**
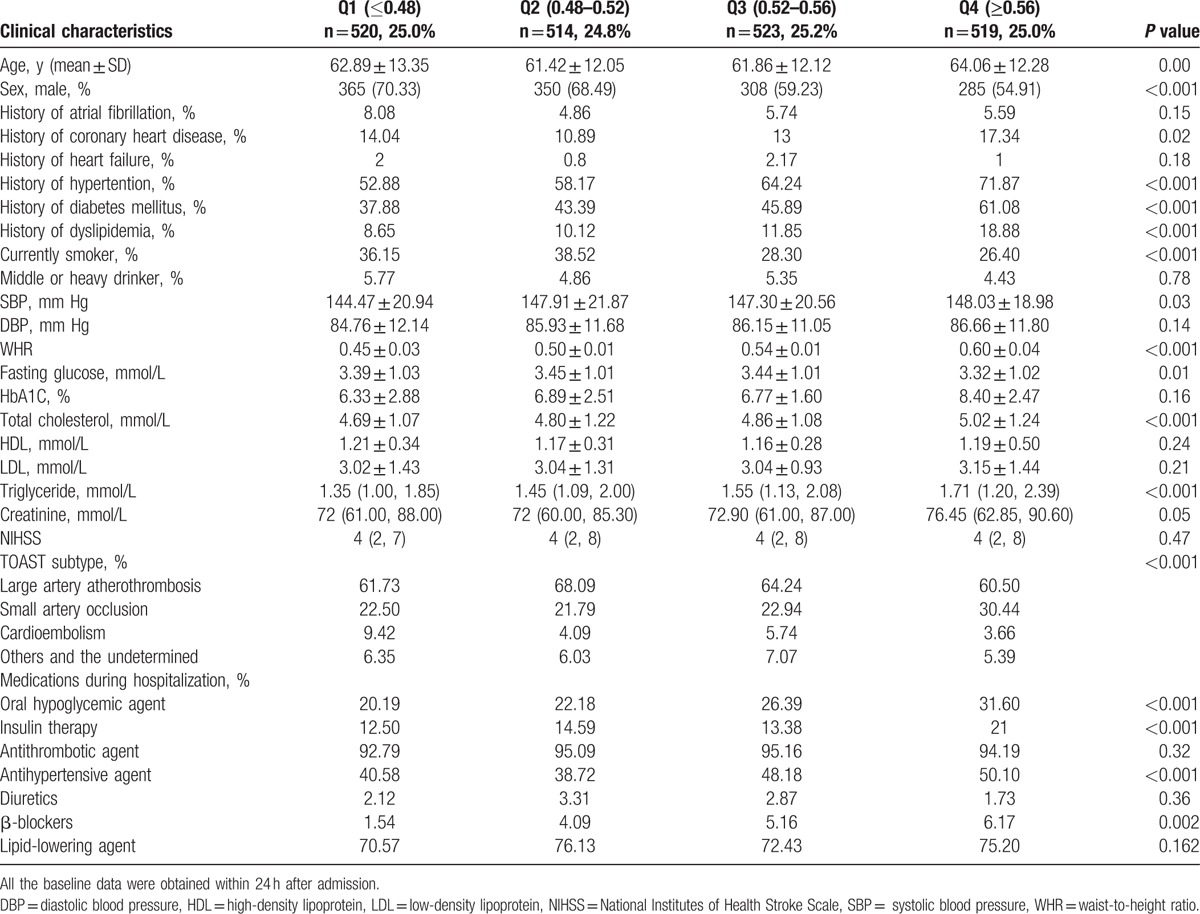
Baseline characteristics of patients with stroke at admission according to different quartiles of waist-to-height ratios.

### 3.1 WHR and functional outcomes

Among the patients with higher grades of functional outcomes, it appeared that the mean age was older, and the percentage of male was lower, the percentages of history of atrial fibrillation, coronary heart disease, and diabetes mellitus were higher. The patients with different functional outcomes were also different in the following characteristics: smoking status, NIHSS score, WHR and serum triglyceride level and high density lipoprotein level, as well as a history of heart failure as well as more in-hospital insulin usage, less antithrombotic and lipid-lowering agent usage (Table 2). After multivariate adjustment, we found that, compared with the first WHR quartile, the fourth quartile was associated with higher grades of mRS scores (second quartile, OR = 1.05, CI: 0.83–1.33; third quartile, OR = 1.10, CI: 0.86–1.40; fourth quartile, OR = 1.38, CI: 1.08–1.77) (Table 3).

**Table 2:**
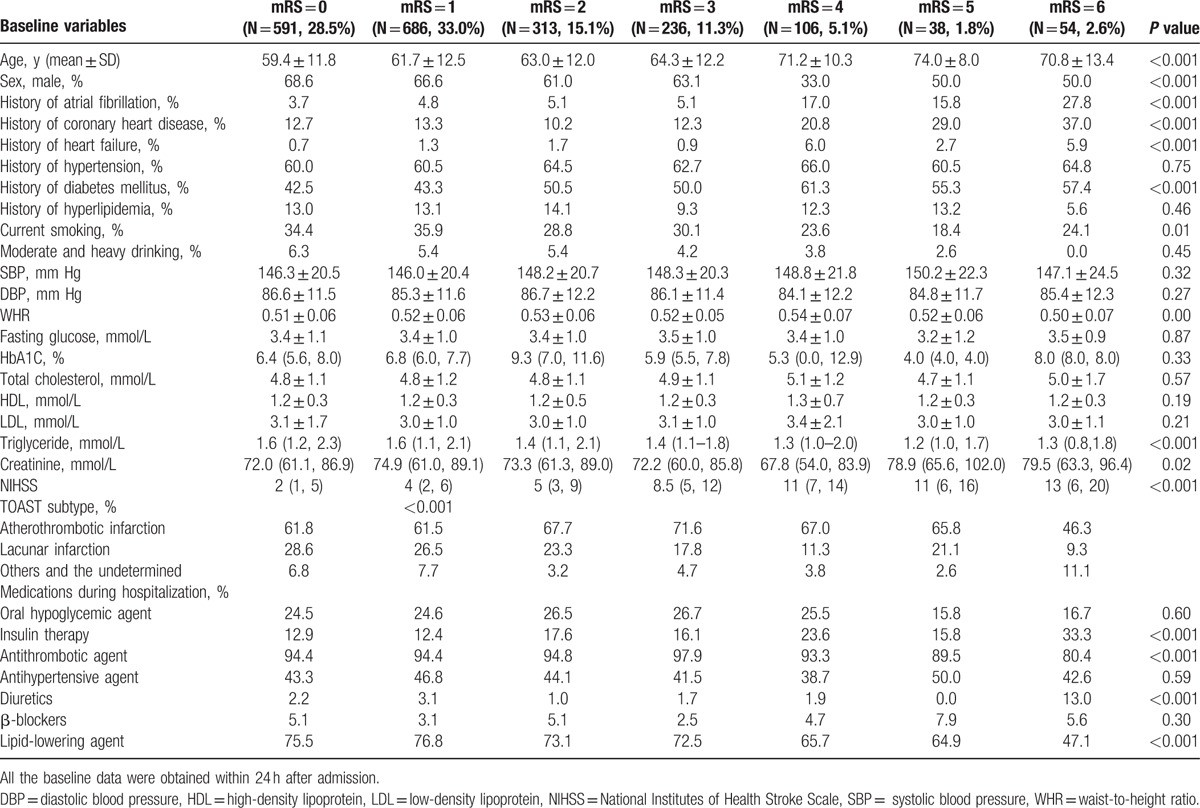
Univariate analysis of waist-to-height ratio and functional outcome.

**Table 3:**
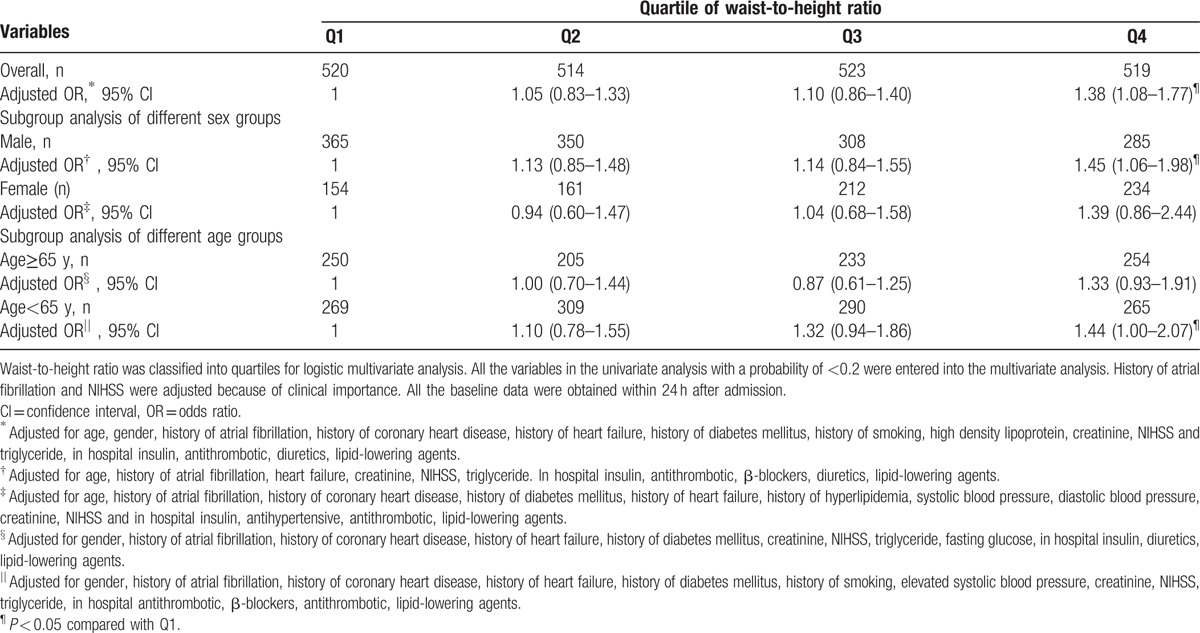
Multivariate analysis of waist-to-height ratio and functional outcome.

The subgroup analysis revealed that in the male subgroup, compared with the first quartile, the fourth WHR quartile was significantly associated with higher grades of mRS scores (fourth quartile, OR = 1.45, CI: 1.06–1.98, *P* = 0.02). However, this association was not observed in the female subgroup. We also did observe in the younger age (<65) subgroup, compared with the first quartile, the fourth WHR quartile was significantly associated with higher grades of mRS scores (fourth quartile, OR = 1.44, CI: 1.00–2.07, *P* = 0.05) (Table 3).

### 3.2 WHR and mortality outcomes

Among the patients who had survived to the 1-year follow-up, the mean age was 61.8 years old, and 63.8% were males. Among the patients who had died before the 1-year follow-up, the mean age was 70.0 years old, and 58.9% were males. The patients with mortality outcomes were more likely than the patients with survival outcomes to have the following characteristics: older, higher NIHSS score, less moderate and heavy drinking as well as history of atrial fibrillation, coronary heart disease, heart failure, diabetes mellitus, as well as more oral hypoglycemic, insulin and diuretics usage, less antithrombotic agent usage (Table 4). After multivariate adjustment, only older age, history of diabetes mellitus, higher systolic and diastolic blood pressure, in-hospital diuretics usage, and higher NIHSS score at admission was associated with mortality outcomes. The WHR was not found to be correlated with mortality outcomes (second quartile, HR = 0.69, CI: 0.46–1.04; third quartile, HR = 0.79, CI: 0.53–1.18; fourth quartile, HR = 0.85, CI: 0.58–1.25) (Table 5).

**Table 4:**
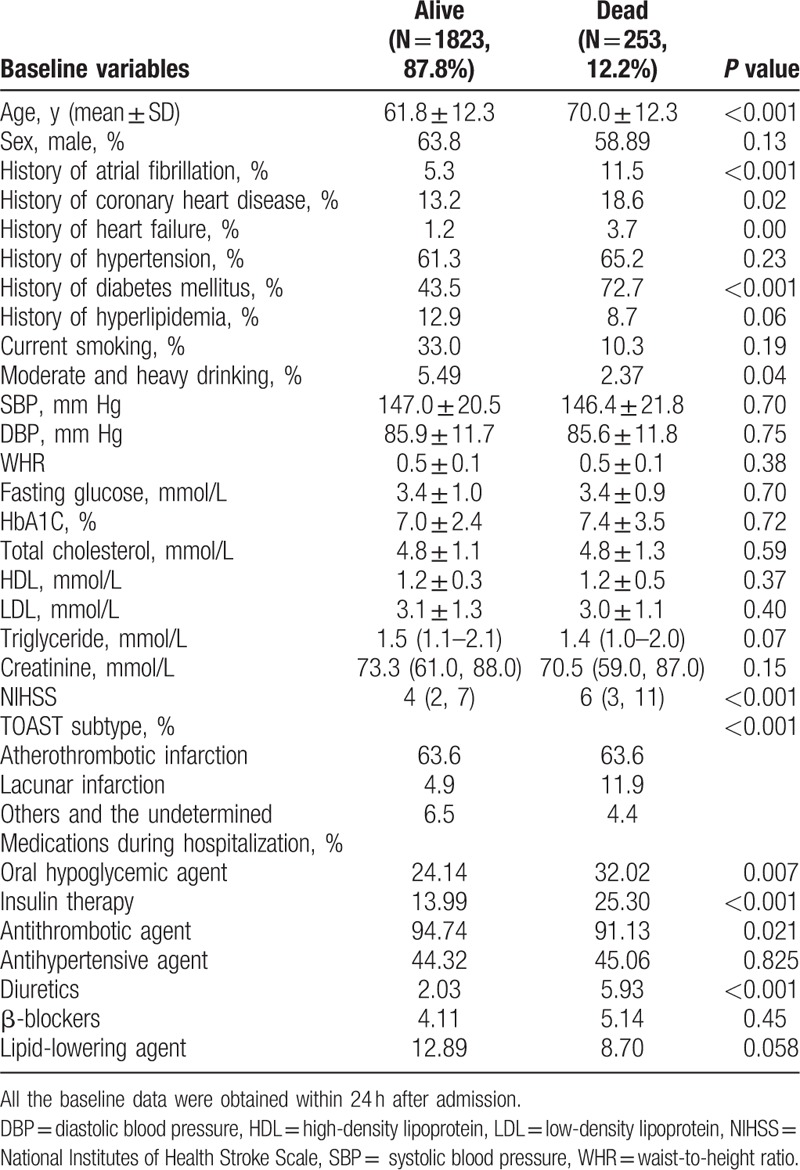
Univariate analysis of waist-to-height ratio and mortality outcome.

**Table 5:**
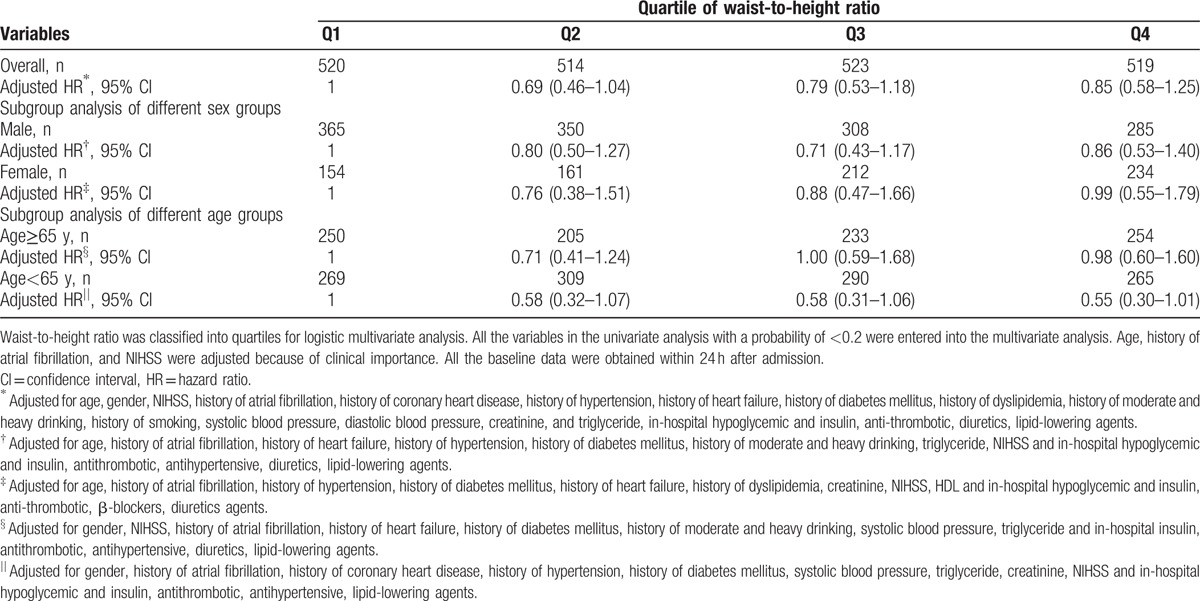
Multivariate analysis of waist-to-height ratio and mortality outcome.

In the subgroup analysis of different sex- and age-based groups, the WHR remained a nonsignificant predictor of mortality outcomes (Table 5).

## 4 Discussion

Our study showed that the WHR was possibly associated with poorer functional outcomes, but unable to predict patients’ mortality outcomes. However, in the subgroup analysis, we found the association was only significant in the male subgroup and in the subgroup with age younger than 65. This result was derived from ACROSS-China, a multicenter, prospective stroke registry, with a sample size of over 2000 participants. Our study was an exploration of the influence of abdominal fat accumulation on stroke prognosis in an Asian population.

Previous work has mainly focused on the relationship between BMI and stroke-related mortality.^[19,20]^ Such work has shown that among stroke patients, overweight or obese patients had a more favorable chance of survival than those who were underweight or of a normal weight, as defined by BMI. However, Danish researchers concluded that there was no obesity paradox after adjusting for selection bias, and no difference in the risk of stroke-related death in the first month was found between different BMI groups.^[5]^ They also discovered that in patients with a higher BMI, the first stroke occurred at a significantly younger age (compared with the time of first occurrence in individuals of a normal weight, stroke occurred 3 years earlier in overweight patients and 6 years earlier in obese patients). Although BMI is widely used, it cannot reveal the distribution of body fat. Therefore, in our study, we performed an analysis using WHR as a substitution.

Waist circumference, the WHpR, and the WHR are all abdominal adiposity indices. The Northern Manhattan Stroke Study showed that abdominal obesity was an independent risk factor for ischemic stroke using the WHpR.^[21]^ The WHpR was also identified by the INTERSTROKE study as 1 of 10 risk factors for stroke, irrespective of the stroke type.^[22]^ However, the practical value of this index was limited because the hip and waist circumferences often change together in response to weight fluctuations. Waist circumference has been considered a proxy for visceral adiposity in studies that used advanced imaging technique, and both WHO and Chinese experts recommend it to be measured regularly.^[23]^ Nevertheless, waist circumference was found to be less predictive of intra-abdominal fat than WHR,^[24]^ and the cutting points were different between man and women. For this reason, the WHR was identified as a better proxy to predict diabetes and cardiovascular disease. A WHR of 0.5 has been recommended as a suitable global boundary value.^[11]^ Similar to our study, a Mexican research discovered that the excess of adiposity reflected by the WHR increased the chance of severe disability after ischemic stroke.^[25]^

Sex differences were investigated in our study because men and women differ in body shape and in brain structure and metabolism,^[26]^, and we did, in fact, detect sex differences in our study. Previous studies have shown that obese men experience a greater risk of stroke than obese women, after adjusting for age, ethnicity, smoking status, hypertension, and diabetes mellitus. BMI was the only measure of obesity and was simply a proxy for general obesity.^[27]^ In our study, only male patients seemed to subject to abdominal fat accumulation, as measured by the WHR. This observation may be explained by the “FFA flow theory.” Women are naturally predisposed to a “pear” body shape, while men are more likely to have an “apple” body shape. Studies have found that “apples” are more likely to store visceral fat than “pears.” From this, we can infer that males might be subject to greater risk than the females. FFAs metabolized by visceral fat drift into the liver, leading to increased insulin resistance^[28]^ and VLDL and triglyceride production.^[29]^ In vivo studies have shown that this effect is more obvious in women than in men.^[30]^ Although the influence of the WHR was not significant in women, it might be due to its smaller sample size of female subgroup in our study. We cannot exclude the possibility that controlling the WHR is not necessary in women, not to mention that weight control is known to be beneficial for the prevention of a wide range of diseases.

We also observed the association of WHR on the functional outcomes of stroke patients was mainly on the younger people (<65 years old). Researchers have found trend toward increasing stroke incidence at younger ages.^[31]^ Also obesity increases risk of ischemic stroke in young adults.^[27,32]^ Whether obesity directly or via intermediate factors affects the incidence and the prognosis of stroke is still uncertain. Prevention of obesity among youth and WHR-control treatment in younger patients is probably relatively more important.

Our study explored the influence of the WHR, an abdominal obesity proxy, on the 1-year functional and mortality outcomes of a relatively large sample of first ischemic stroke patients. We did not observe an inverse relationship between the WHR and mortality outcomes. Instead, we found a potential harmful effect of the WHR on poststroke dependency, especially on man and younger patients. However, our study did have some limitations. First, patient compliance with the prescribed treatment and rehabilitation regimens could have affected functional outcomes. Second, the ACROSS-China study's selection of participating hospitals was through convenience, and these hospitals were located in urban regions of China. Third, patients’ diet could influence the outcome of our research, but we were lack of the diet information, which is another limitation of our study. Forth, self-reported data such as the functional state through telephone was less accurate than face-to-face examination. Fifth, the characteristics of the participants in our study were similar to those lost in follow-up, but the proportion of diabetes mellitus was higher among the participants in our study. So this selection bias may affect the final results. And 1 year's follow-up might influence the accuracy of the outcome data. Future studies should focus on how to maintain a good body shape and measure the beneficial influence of shaping the body.

## 5 Conclusions

Our study indicated that abdominal fat accumulation may be associated with functional recovery after stroke, and is not associated with mortality after stroke. Compared with the lowest quartile, the highest quartile of WHR at admission was possibly associated with worse postacute ischemic stroke functional recovery. Providing instructions to help patients manage not only their traditional risk factors but also their WHR is of clinical value. Unlike age or disease severity at admission, the WHR is a controllable prognostic factor and should not be neglected by physicians. Our finding agrees to some extent with the public health slogan “Make your waist less than half of your height.”
